# Supplementary material for: Outcomes of Octogenarians with Primary Malignant Cardiac Tumors: National Cancer Database Analysis
Source: J Clin Med. 2022 Aug 20;11(16):4899. doi: 10.3390/jcm11164899 (PMC9410046; doi:10.3390/jcm11164899)

Supplementary Table S1: Criteria and outcomes among surgically treated patients

	Overall	Younger	Septuagenarian	Octogenarian	p
n	432	374	40	18	
Age (median [IQR])	50.50 [37.00, 63.00]	46.50 [36.00, 58.00]	75.00 [72.00, 76.25]	83.00 [82.00, 84.00]	<0.001
Female (%)	210 (48.6)	178 (47.6)	21 (52.5)	11 (61.1)	0.467
Race (%)					0.219
• White	358 (83.8)	304 (82.4)	37 (92.5)	17 (94.4)	
• Black	45 (10.5)	44 (11.9)	1 (2.5)	0 (0.0)	
• Others	24 (5.6)	21 (5.7)	2 (5.0)	1 (5.6)	
Insurance status					<0.001
• No insurance/Unknown	35 (8.1)	34 (9.1)	1 (2.5)	0 (0.0)	
• Private	260 (60.2)	251 (67.1)	6 (15.0)	3 (16.7)	
• Governmental	137 (31.7)	89 (23.8)	82.5)	15 (83.3)	
Median income quartile (%)					0.348
• Less than $40,227	66 (16.1)	58 (16.4)	4 (10.0)	4 (22.2)	
• $40,227 - $50,353	79 (19.2)	67 (19.0)	7 (17.5)	5 (27.8)	
• $50,354 - $63,332	104 (25.3)	94 (26.6)	9 (22.5)	1 (5.6)	
• $63,333 or more	162 (39.4)	134 (38.0)	50.0)	8 (44.4)	
Urban/Rural counties (%)					0.72
• Metropolitan	356 (85.8)	305 (85.0)	34 (89.5)	17 (94.4)	
• Urban	54 (13.0)	49 (13.6)	4 (10.5)	1 (5.6)	
• Rural	5 (1.2)	5 (1.4)	(0.0)	0 (0.0)	
No high school graduate quartile (%)¶					0.113
• 17.6% or more	78 (19.0)	74 (21.0)	3 (7.5)	1 (5.6)	
• 10.9% - 17.5%	102 (24.8)	84 (23.8)	11 (27.5)	7 (38.9)	
• 6.3% - 10.8%	105 (25.5)	93 (26.3)	9 (22.5)	3 (16.7)	
• Less than 6.3%	126 (30.7)	102 (28.9)	42.5)	7 (38.9)	
Great circle distance (miles; median [IQR]) ¶¶	18.50 [7.00, 46.65]	19.50 [7.60, 49.20]	13.90 [5.38, 38.30]	10.30 [3.95, 22.05]	0.052
Facility type (%)					<0.001

• Academic/integrated	211 (48.8)	173 (46.3)	26 (65.0)	12 (66.7)	
• Community	97 (22.5)	77 (20.6)	14 (35.0)	6 (33.3)	
• Unknown	124 (28.7)	124 (33.2)	(0.0)	0 (0.0)	
CDCC (0 or 1 / 2 or 3) (%) *	388/44 (89.8/10.2)	341/33 (91.2/8.8)	34/6 (85.0/15.0)	13/5 (72.2/27.8)	0.02
Year of diagnosis (median [IQR])	2010.00 [2007.00, 2014.00]	2010.00 [2007.00, 2013.00]	2013.00 [2009.00, 2014.00]	2012.50 [2008.25, 2015.00]	0.095
Histology (%)					0.027
• Angiosarcoma	145 (33.6)	129 (34.5)	11 (27.5)	5 (27.8)	
• Fibrosarcoma	23 (5.3)	21 (5.6)	0 (0.0)	2 (11.1)	
• Giant cell sarcoma	29 (6.7)	23 (6.1)	5 (12.5)	1 (5.6)	
• Leiomyosarcoma	31 (7.2)	26 (7.0)	3 (7.5)	2 (11.1)	
• Liposarcoma	6 (1.4)	3 (0.8)	3 (7.5)	0 (0.0)	
• Malignant fibrous histiocytoma (MFH)	10 (2.3)	10 (2.7)	0 (0.0)	0 (0.0)	
• MPNST	3 (0.7)	3 (0.8)	0 (0.0)	0 (0.0)	
• Myxosarcoma	18 (4.2)	17 (4.5)	1 (2.5)	0 (0.0)	
• Osteosarcoma	17 (3.9)	17 (4.5)	0 (0.0)	0 (0.0)	
• Others/Unclassified	112 (25.9)	95 (25.4)	11 (27.5)	6 (33.3)	
• Rhabdomyosarcoma (RMS)	14 (3.2)	8 (2.1)	4 (10.0)	2 (11.1)	
• Synovial sarcoma	24 (5.6)	22 (5.9)	(5.0)	0 (0.0)	
Grade (poorly differentiated/anaplastic) (%)	251 (58.1)	212 (56.7)	26 (65.0)	13 (72.2)	0.277
Tumor size (in mm; median [IQR])	55.00 [43.00, 75.00]	57.00 [44.50, 78.50]	50.00 [34.00, 66.50]	50.00 [41.00, 60.00]	0.247
Analytic stage group (%)					0.02
• Stage 0/I/x	212 (49.1)	184 (49.2)	21 (52.5)	7 (38.9)	
• Stage II	71 (16.4)	56 (15.0)	7 (17.5)	8 (44.4)	
• Stage III/IV	149 (34.5)	134 (35.8)	30.0)	3 (16.7)	
Surgery (%)	432 (100.0)	374 (100.0)	40 (100.0)	18 (100.0)	NA
Radiation (%)					0.165
• No	343 (79.4)	296 (79.1)	31 (77.5)	16 (88.9)	
• Yes	73 (16.9)	66 (17.6)	7 (17.5)	0 (0.0)	

• Unknow	16 (3.7)	12 (3.2)	(5.0)	2 (11.1)	
Chemotherapy (%)					<0.001
• No	171 (39.6)	136 (36.4)	25 (62.5)	10 (55.6)	
• Yes	219 (50.7)	208 (55.6)	10 (25.0)	1 (5.6)	
• Unknow	42 (9.7)	30 (8.0)	5 (12.5)	7 (38.9)	
30-day mortality (Alive/Dead) (%)	392/39 (91.0/9.0)	347/26 (93.0/7.0)	34/6 (85.0/15.0)	11/7 (61.1/38.9)	<0.001

¶This item provides a measure of the number of adults age 25 or older in the patient's zip code who did not graduate from high school, and is categorized as equally proportioned quartiles among all US zip codes.

¶¶The "great circle" distance in miles between the patient's residence and the hospital that reported the case.

* CDCC: Charlson/Deyo comorbidity condition, MPNST: Malignant peripheral nerve sheath tumors

Supplementary Table S2: Independent predictors of 30 day mortality among the surgical cohort (n=39/432)

Variable	Units	Univariate analysis<br>Odds Ratio (95% CI) p-value	Multivariable analysis<br>Odds Ratio (95% CI) p-value
Age	Younger	Ref	Ref
	Septuagenarian	2.36 [0.91;6.12] 0.079	2.22 [0.84;5.82] 0.106
	Octogenarian	**8.49 [3.04;23.74] <0.01**	**7.31 [2.55;20.94] <0.01**
Sex	Male	Ref	
	Female	1.13 [0.59;2.18] 0.71	
Race	White	Ref	
	Black	1.42 [0.52;3.87] 0.50	
	Others	2.39 [0.76;7.49] 0.14	
Insurance	No/Unknown	Ref	
	Private	0.61 [0.20;1.92] 0.40	
	Governmental	1.02 [0.32;3.28] 0.97	
Median income quartile	Less than $40,227	Ref	
	More than $40,227	1.02 [0.41;2.55] 0.96	
Urban/Metropolitan	Urban	Ref	
	Metropolitan	0.73 [0.25;2.15] 0.57	
	Rural	0.00 [0.00;Inf] 0.99	
CDCC ¶	0/I	Ref	Ref
	II/III	**3.06 [1.35;6.96] <0.01**	**2.46 [1.03;5.86] 0.042**
Year of diagnosis		0.97 [0.89;1.06] 0.53	
Histology	Others	Ref	
	Angiosarcoma	1.42 [0.72;2.78] 0.31	
Analytic stage	Stage 0/I/x	Ref	
	Stage II	1.28 [0.54;3.07] 0.58	
	Stage III/IV	0.89 [0.42;1.88] 0.75	
Facility type	Community	Ref	

	Academic/Integrated	1.17 [0.54;2.54] 0.69	

¶ CDCC: Charlson/Deyo comorbidity condition

Figure S1: Cumulative death from Any Cause Among different age categories inpatients who underwent surgery

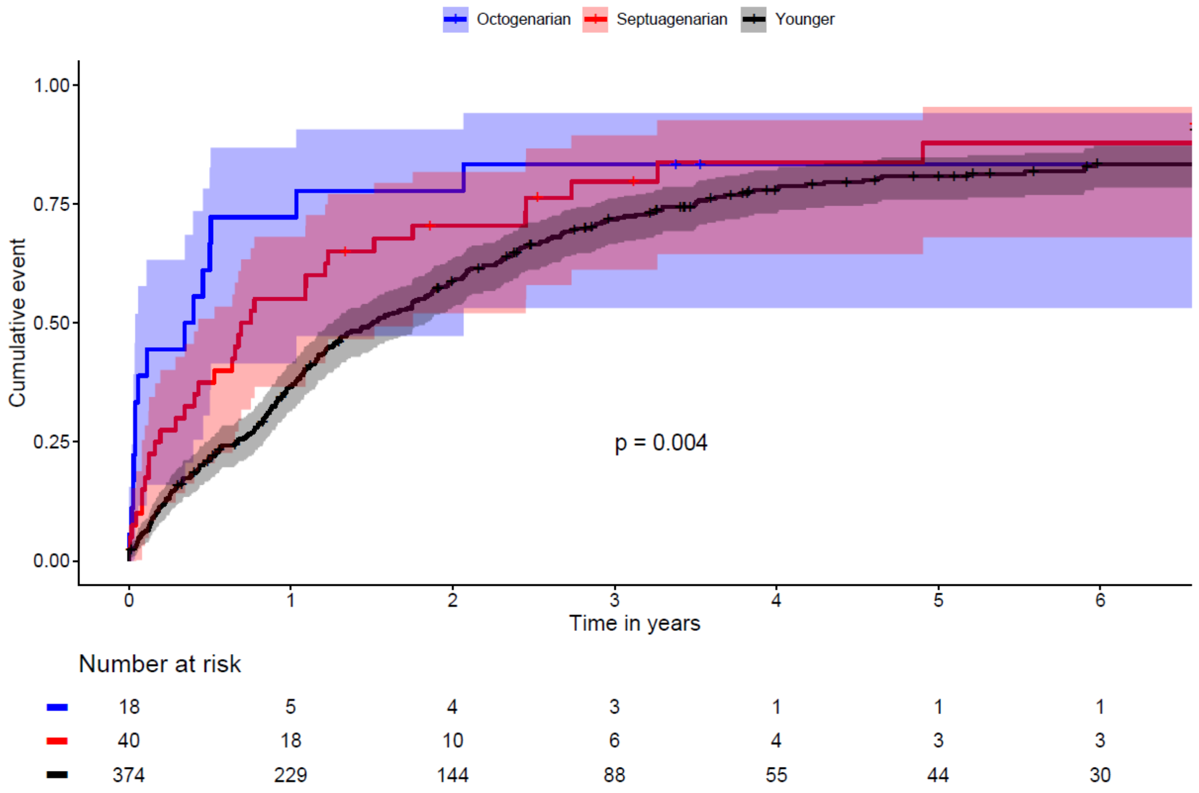

	Median OS (months)	2 years OS	3 years OS	5 years OS
Overall	15.74 (13.90-20.11)	39.2	27.0	18.3
Octogenarian	4.45 (0.46-24.80)	22.20	16.70	16.70
Septuagenarian	8.67 (5.13-21.03)	29.60	20.30	12.20
Younger	18.10 (15.11-21.95)	41.10	28.20	19.20

Figure S2: Annual 30-day mortality percent among different age categories in the surgical cohort. Categories with 100% mortality had ≤2 cases/year

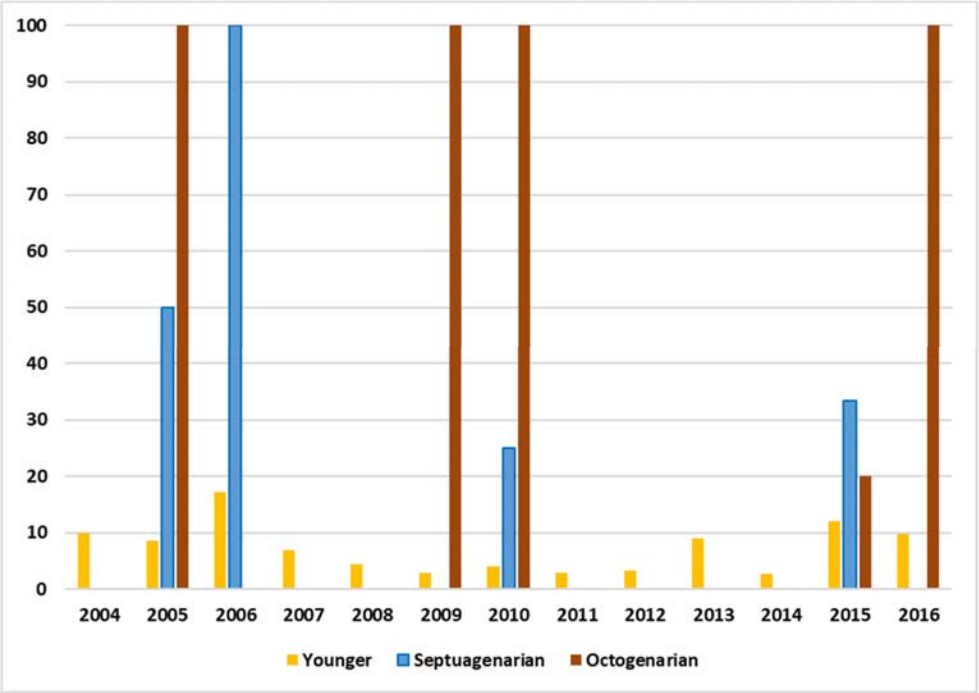

Supplement: Supplementary file 1 [file jcm-11-04899-s001.zip › jcm-1856658-supplementary.pdf]
